# The Protein Interactome of *Streptococcus pneumoniae* and Bacterial Meta-interactomes Improve Function Predictions

**DOI:** 10.1128/mSystems.00019-17

**Published:** 2017-06-06

**Authors:** S. Wuchty, S. V. Rajagopala, S. M. Blazie, J. R. Parrish, S. Khuri, R. L. Finley, P. Uetz

**Affiliations:** a Department of Computer Science, University of Miami, Coral Gables, Florida, USA; b Center for Computational Science, University of Miami, Coral Gables, Florida, USA; c Sylvester Comprehensive Cancer Center, University of Miami, Miami, Florida, USA; d Department of Biology, University of Miami, Coral Gables, Florida, USA; e J Craig Venter Institute, Rockville, Maryland, USA; f Center for Molecular Medicine and Genetics, Wayne State University School of Medicine, Detroit, Michigan, USA; g Center for the Study of Biological Complexity, Virginia Commonwealth University, Richmond, Virginia, USA; USDA—Agricultural Research Service, Boyce Thompson Institute for Plant Research, Cornell University

**Keywords:** functional prediction, protein-protein interactions

## Abstract

The functions of roughly a third of all proteins in *Streptococcus pneumoniae*, a significant human-pathogenic bacterium, are unknown. Using a yeast two-hybrid approach, we have determined more than 2,000 novel protein interactions in this organism. We augmented this network with meta-interactome data that we defined as the pool of all interactions between evolutionarily conserved proteins in other bacteria. We found that such interactions significantly improved our ability to predict a protein’s function, allowing us to provide functional predictions for 299 *S. pneumoniae* proteins with previously unknown functions.

**IMPORTANCE** Identification of protein interactions in bacterial species can help define the individual roles that proteins play in cellular pathways and pathogenesis. Very few protein interactions have been identified for the important human pathogen *S. pneumoniae*. We used an experimental approach to identify over 2,000 new protein interactions for *S. pneumoniae*, the most extensive interactome data for this bacterium to date. To predict protein function, we used our interactome data augmented with interactions from other closely related bacteria. The combination of the experimental data and meta-interactome data significantly improved the prediction results, allowing us to assign possible functions to a large number of poorly characterized proteins.

## Abstract (precis)

Identification of protein interactions in bacterial species can help define the individual roles that proteins play in cellular pathways and pathogenesis. Very few protein interactions have been identified for the important human pathogen *S. pneumoniae*. We used an experimental approach to identify over 2,000 new protein interactions for *S. pneumoniae*, the most extensive interactome data for this bacterium to date. To predict protein function, we used our interactome data augmented with interactions from other closely related bacteria. The combination of the experimental data and meta-interactome data significantly improved the prediction results, allowing us to assign possible functions to a large number of poorly characterized proteins.

## INTRODUCTION

The discovery of protein interactions in bacteria has been a powerful way to understand how proteins function in cellular pathways and pathogenesis. However, only a few studies have set out to identify the protein interactions in bacterial species, including *Campylobacter jejuni* (1), *Treponema pallidum* (2), *Synechocystis* sp. (3), *Mycobacterium tuberculosis* (4), *Mesorhizobium loti* (5), and, recently, *Escherichia coli*, *Mycoplasma pneumoniae* (6–10), and *Helicobacter pylori* (11). In addition, a partial interactome is available for *Bacillus subtilis* (12). Most of these studies used yeast two-hybrid (Y2H) screening technology (13), mapping pairwise interactions between proteins. A few studies systematically identified the composition of protein complexes using affinity purification and mass spectrometry (AP/MS) (7–10). In all cases, the interactome data have been shown to be useful for identifying protein function, for understanding cellular pathways, and even for identifying drug targets and other points for intervention in the case of pathogens.

Despite the proven value of large-scale protein interaction data, relatively little is known about the interactome of the human pathogen *Streptococcus pneumoniae*, which causes millions of episodes of serious pneumococcal disease each year, including more than 1.8 million deaths worldwide in children under 5 years of age (14, 15). A better understanding of the interactome of *Streptococcus* bacteria would contribute to our ability to therapeutically intervene and to find novel antibiotics (or explanations for resistance to them).

More than 600 (29%) of the 2,109 proteins of *S. pneumoniae* are still uncharacterized, while many more proteins have only very general or predicted annotations, such as “membrane protein” or “ABC transporter,” without known specificity (per the UniProt reference proteome data available in June 2016). The fraction of uncharacterized proteins in *S. pneumoniae* is similar to that in other bacteria, reflecting a need for protein function studies in bacteria in general. For instance, our study of the *Treponema pallidum* interactome (2) has led to the characterization of several proteins of previously “unknown” function such as YbeB (now RsfS), a ribosomal silencing factor (16), and TP0658 (FliW), a regulator of flagellin mRNA translation and assembly (17). While protein interactions are considered the backbone for numerous cellular activities (18), such interactions remain unknown in most species or uncharacterized even in many model organisms. As for *S. pneumoniae*, relatively few protein interactions have been determined and no proteome-wide screens have been reported so far.

Simple statistical methods such as the “majority rule” can predict the function of uncharacterized proteins by considering the majority of functions of their interaction partners (19–21). For example, if most of its interaction partners are ribosomal proteins, the function of an “unknown” protein is predicted to be likely “ribosomal” as well. While this approach is promising, the prediction accuracy depends on the number, completeness, and reliability of the available protein interaction networks and other functional information concerning the interacting proteins (22).

We have previously shown that use of multiple variants of the Y2H approach can produce more-comprehensive coverage of interactions for any set of proteins, allowing discovery of many protein interactions that would be missed with the use of one experimental approach alone (23–25). As a consequence, we decided to apply two complementary Y2H approaches to determine more than 2,000 protein-protein interactions (PPIs) between *S. pneumoniae* proteins. This allowed us to predict functions for many poorly characterized proteins by connecting them to known proteins and pathways. To further enhance our ability to predict protein functions, we augmented the experimentally derived *S. pneumoniae* protein network with interactions predicted from other bacteria. Notably, we found that prediction accuracy increased with the addition of a protein interaction network that combined the experimental data with the bacterial meta-interactome. We further show that such prediction characteristics increase as a function of a given protein’s degree (number of interactions) in the original and augmented networks. The predictions appeared functionally consistent in comparisons of results in the original and augmented networks. Finally, we utilize our bacterial meta-interactome to predict the functions of 299 poorly characterized genes in *S. pneumoniae*.

## RESULTS

### The binary *S. pneumoniae* interactome.

**FIG 1:**
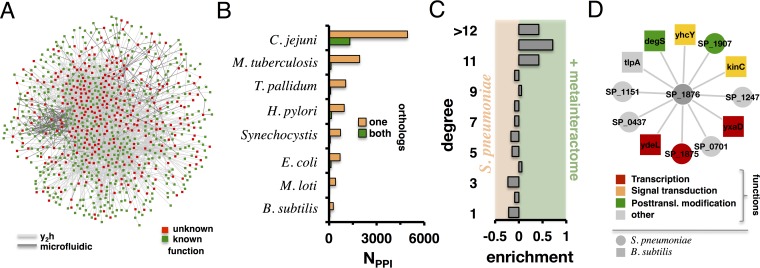
Characteristics of bacterial interactomes. (A) We schematically show the combined network of *S. pneumoniae* protein interactions detected by yeast two-hybrid (light gray edges) or microfluidics (dark gray edges). Proteins with known function are colored green; the functions were unknown (red nodes) for 342 (37.2%) of the 918 proteins in the network. (B) To augment our network of *S. pneumoniae* protein interactions, we utilized interactomes from other bacteria. The numbers of PPIs are shown for interactions where both proteins have (green) or only one protein has (orange) *S. pneumoniae* orthologs. (C) Considering all genes in *S. pneumoniae* without known functions, we calculated the number of interaction partners (degree) in the original *S. pneumoniae* network (left) and in the network augmented with the meta-interactome (right). Proteins with higher degrees mostly benefited from the addition of interologs. (D) As an example, SP_1876 interacted with 6 *S. pneumoniae* proteins (circles) plus another 6 *B. subtilis* proteins (squares) in the augmented network. As a result, functions of interaction partners of SP_1876 mostly revolve around transcription, signal transduction, and posttranslational (Posttransl.) modifications (based on EggNOG; see Materials and Methods and Discussion).

Using two different Y2H approaches (1, 6), we determined a total of 2,045 binary interactions between 820 proteins of *S. pneumoniae*, a Gram-positive bacterium with 2,109 predicted open reading frames (ORFs) (see Table S1 in the supplemental material). We screened all available proteins against each other using a Gal4-based Y2H system. To gain more insight into protein functions and to increase coverage of poorly characterized genes, we also screened 360 hypothetical proteins using a LexA-based system (see Table S2 and Materials and Methods for details). An additional set of 322 protein interactions were previously determined by a microfluidic high-throughput assay (26, 27) (all interactions are provided in Table S3). Combining these three sets of experimentally determined binary protein interactions for *S. pneumoniae* resulted in a network of 2,353 interactions between 918 proteins, covering 43% of the *S. pneumoniae* proteome (Fig. 1A).

10.1128/mSystems.00019-17.1 TABLE S1  All available ORF clones were obtained from the Pathogen Functional Genomics Resource Center (PFGRC) at the J. Craig Venter Institute (JCVI); all clones were used as baits and preys. Download TABLE S1, XLSX file, 0.1 MB. Copyright © 2017 Wuchty et al. 2017 Wuchty et al. This content is distributed under the terms of the Creative Commons Attribution 4.0 International license.

10.1128/mSystems.00019-17.2 TABLE S2  All bait clones screened by the LexA Y2H system. Download TABLE S2, XLSX file, 0.1 MB. Copyright © 2017 Wuchty et al. 2017 Wuchty et al. This content is distributed under the terms of the Creative Commons Attribution 4.0 International license.

10.1128/mSystems.00019-17.3 TABLE S3  All PPIs found by both the Gal4 and LexA screens; reciprocal hits represent PPIs that were found when bait clones were used as prey and vice versa. Download TABLE S3, XLSX file, 0.1 MB. Copyright © 2017 Wuchty et al. 2017 Wuchty et al. This content is distributed under the terms of the Creative Commons Attribution 4.0 International license.

### Protein function prediction.

The binary interaction network can be used to suggest functions for many poorly characterized proteins by connecting them to proteins or pathways with known function (see below). However, a limitation of the experimentally derived network for protein function prediction is that it contains a large number of functionally uncharacterized proteins. In the combined network, the functions of 342 of 918 (37.2%) proteins were unknown (Table S1) (28). Given that a large fraction of bacterial proteins lack functional annotations, results determined using the majority rule approach to infer protein function are not always informative. However, we hypothesized that the combination of interactions that involve conserved proteins from many bacterial species into a meta-interactome may significantly improve our ability to predict functions of unknown proteins in *S. pneumoniae*. To build the meta-interactome, we included experimentally derived protein interactions from *C. jejuni*, *M. loti*, *Synechocystis*, *B. subtilis*, *T. pallidum*, *H. pylori*, *E. coli*, and *M. tuberculosis*, whose interactomes have been determined on a large scale (1–6, 11, 12). We defined the bacterial meta-interactome as the union of all interactions from other bacteria that involve at least one orthologous protein in *S. pneumoniae*. Although such interactions may have only one ortholog, we surmise that nonconserved proteins that have known functions could point to the functions of their conserved interaction partners, including proteins with unknown functions in *S. pneumoniae*. Utilizing the InParanoid script (29), we determined interactions between proteins in the underlying bacterial interaction data that had at least one ortholog in *S. pneumoniae*; as expected, species with more available interactome data, such as *C. jejuni* and *M. tuberculosis*, contributed the greatest number of PPIs to the meta-interactome (Fig. 1B). To assess the impact of such interactions on the network neighborhood of proteins with unknown function, we determined the number of interaction partners in the binary protein interaction network of *S. pneumoniae* as well as in a network that was augmented with the bacterial meta-interactome. As shown in Fig. 1C, we observed that sparsely connected proteins with unknown function appeared less frequently in the augmented network. As an example, we focused on the hypothetical protein SP_1876, which in the original *S. pneumoniae* network had six binding partners with no predominant functional annotation (Fig. 1D; Table 1). In the augmented network, however, SP_1876 had six additional interactions, all inferred from the *B. subtilis* ortholog of SP_1876, segregation and condensation protein A (*ScpA*). This protein participates in the SMC condensin complex in *B. subtilis*, organizing and compacting chromosomes during growth (30–32). While ScpA interacting proteins did not have orthologs in *S. pneumoniae*, they carried functional annotations and interactions revolving around transcription and signal transduction. As a result, we observed that the majority of functions in the network neighborhood of SP_1876 featured transcriptional activities (Fig. 1D; Table 1).

**TABLE 1:** Meta-interactome data can improve functional predictions—an example^a^

SP_1876 interacts with:	Description	Function
**SP_1151**	Exonuclease	Replication
**SP_0437**	Glutamyl-tRNA(Gln) amidotransferase	Translation
**SP_1247**	Chromosome segregation protein	Cell cycle
**SP_1875**	Segregation and condensation protein B	Transcription
**SP_0701**	Orotidine 5′-phosphate decarboxylase	Nucleotide transport
**SP_1907**	Chaperonin	Posttranslational modification
KinC	Sporulation kinase C	Signal transduction
YdeL	HTH-type transcriptional regulator	Transcription
YxaD	HTH-type transcriptional regulator	Transcription
DegS	Signal transduction histidine-protein kinase/phosphatase	Posttranslational modification
YhcY	Sensor histidine kinase	Signal transduction
TlpA	Methyl-accepting chemotaxis protein	Inorganic ion transport

a A protein of unknown function interacts with 6 proteins in our primary Y2H data set (bold), but addition of meta-interactions from other species is required for indication of a role in transcription, signal transduction, and posttranslational modifications. Locus and protein names are from UniProt (46) and KEGG (47); annotations and functions are from EggNOG (24).

**FIG 2:**
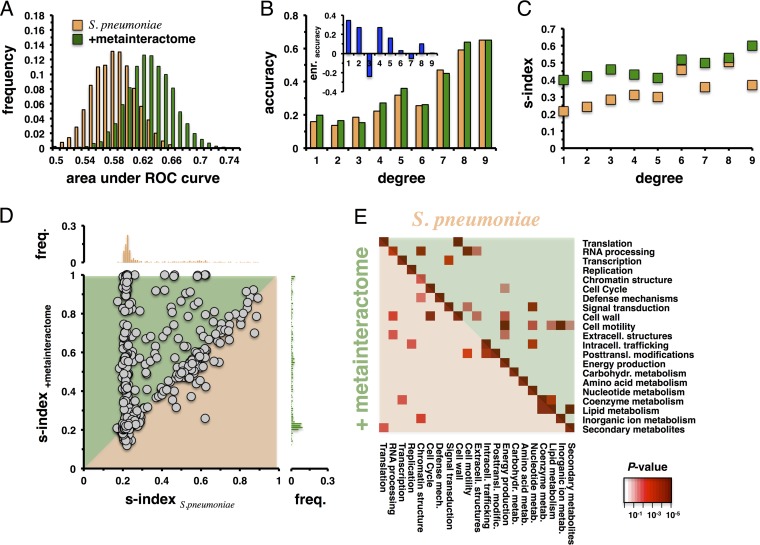
Impact of the bacterial meta-interactome on protein function prediction. (A) To assess the quality of our classification procedure, we randomly sampled 20% of all functionally annotated proteins in *S. pneumoniae* and utilized the remainder to predict their functions. To measure prediction quality, we calculated the area under the ROC curve, suggesting that the addition of the bacterial meta-interactome allowed better functional prediction (*P* < 10^−50^; Student’s *t* test). (B) We calculated the fraction of correctly predicted protein functions as a function of the degree in the original protein interaction network of *S. pneumoniae*. The inset shows the enrichment (enr.) of accuracy (lg2 for the fraction of correctly predicted functions in the original network over the fraction for the augmented network) for each degree, showing that the prediction for proteins with a low degree was improved by adding the meta-interactome. (C) Considering the s-index, predictions of functions appeared more homogeneous with respect to the meta-interactome and increasing degree values. (D) We considered all randomized samples and calculated the mean s-indices of each gene in both the original *S. pneumoniae* network and the augmented network. In the scatterplot, the homogeneity of the functional prediction of the majority of genes benefitted from inclusion of the bacterial meta-interactome. freq., frequency. (E) In each sample, we determined the most probable function for each gene. Counting the occurrence of transitions between such functions in the original *S. pneumoniae* network and the augmented network, we largely found that the functions predicted in the original network corresponded to the same functions in the augmented network.

To investigate the functional predictive power of our initial network of experimentally determined interactions in *S. pneumoniae*, we randomly picked 80% of all functionally annotated proteins 1,000 times to predict the functions of the remaining 20% in each random run. In particular, we utilized functional annotations from the EggNOG database (28). Using a stochastic model (19), we accounted for the observation that an interaction may have been detected multiple times in our screens (see Materials and Methods). As a result, every protein is represented by a profile that reflects the probability of having a certain function. Applying different probability thresholds corresponding to the presence of a functional annotation, we determined receiver operating characteristic (ROC) curves and evaluated the corresponding area under the curve as a measure of the prediction quality (33) (Fig. 2A). Augmenting the experimental protein interaction network of *S. pneumoniae* with interactions from the bacterial meta-interactome, we considered interactions that had at least one interacting protein with a functionally annotated ortholog in *S. pneumoniae* and an interacting counterpart that was at least functionally annotated in the corresponding organism. Focusing on the same, previously sampled sets of proteins, we predicted the functions of the corresponding 20% by utilizing the augmented network. We observed a significant shift toward increased values of the area under the ROC curve (*P* = <10^−50^; Student’s *t* test), suggesting that the augmentation of the original network with interactions from other bacteria significantly improved the quality of functional predictions (Fig. 2A). We next calculated the fraction of correctly predicted protein functions in the random samples as a function of the degree in the protein interaction networks. Figure 2B suggests that prediction accuracy is related to the number of interaction partners of a given protein. As shown in the inset in Fig. 2B, we compared prediction results obtained using the original protein interaction network with results obtained by adding the bacterial meta-interactome. We observed that the addition of the bacterial meta-interactome significantly increased the prediction accuracy for proteins that had a low number of interaction partners in the original *S. pneumoniae* network. Since each protein is represented by a profile of function-specific probabilities, we calculated the Simpson s-index (34) as a measure of the heterogeneity of such probabilities (Fig. 2C). Such a measure tends to correspond to a value of 1 if a single function dominates the distribution of fractions (i.e., has a high probability). In turn, the s-index value approaches 0 if probabilities are equally distributed. While s-index values increased with higher degree values, we observed that profiles obtained with the augmented network had higher s-indices than profiles obtained with the original protein interaction network in *S. pneumoniae*. Since our sampling approach randomly picks a subset of proteins and predicts functions based on the remaining proteins in both the original interaction network of *S. pneumoniae* and the augmented network, we directly compared the effects of the impact of the augmented network on the homogeneity of functional prediction. As shown in Fig. 2D, we calculated the mean s-indices of each protein; the results suggested that functional predictions of a majority of proteins benefitted from the addition of the bacterial meta-interactome. Our data also allowed us to determine if the impact of the meta-interactome changed functional predictions in comparisons of results from the original and augmented networks. Assigning each gene in a given sample the most probable function in the original network and the augmented network, we determined the rates with which genes changed predicted classes in the different networks. Figure 2E suggests that functions that were predicted in the original network of *S. pneumoniae* corresponded to the same class in the augmented network.

**FIG 3:**
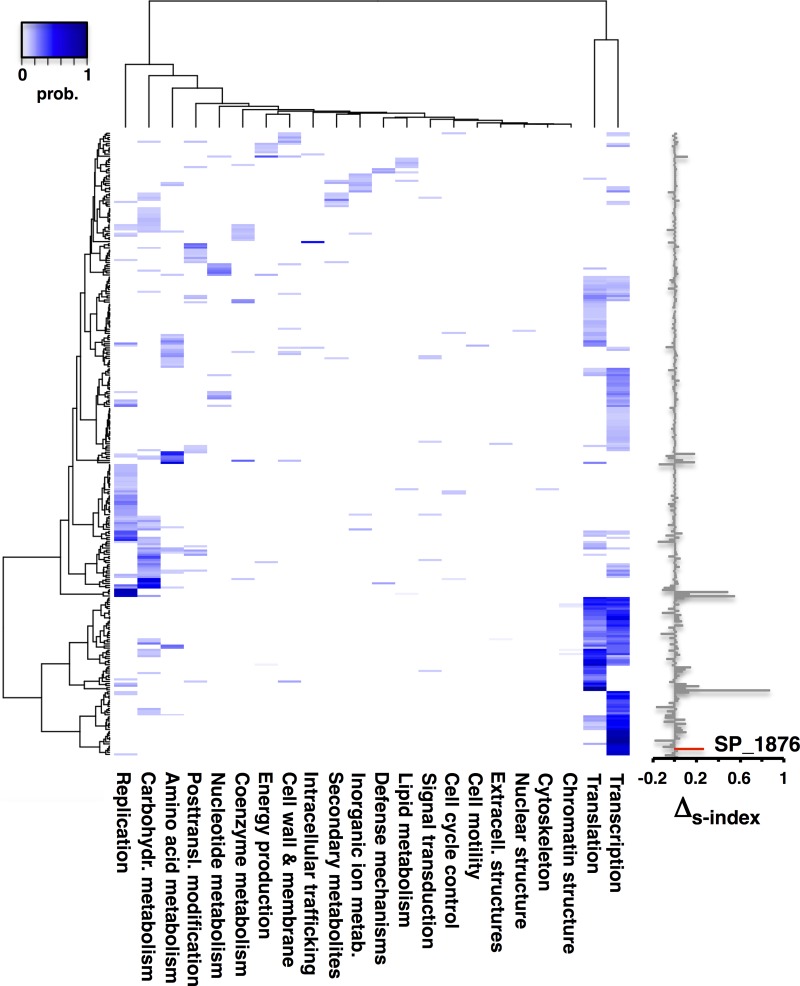
Functional prediction of unknown proteins in *S. pneumoniae*. Augmenting the network of protein interactions of *S. pneumoniae* with interactions of other bacteria, we predicted the functions of 299 proteins with unknown or poorly characterized functions (FDR = <0.05). We annotated each protein with the difference in the s-index value, deducting the corresponding value in the original network of interactions in *S. pneumoniae* and the value in the augmented network. For example, SP_1876 had a 66% chance (FDR = <0.05) of being involved in transcriptional activities. Scores for each protein and the corresponding functional prediction are provided in Table S4.

On the basis of our observations indicating that interactions from other bacteria can have a considerable benefit with respect to our ability to predict functions, we applied our approach to the functional prediction of 342 poorly characterized or previously unknown *S. pneumoniae* proteins. We determined the probability that a given protein has a particular function, and we assessed the significance of our predictions by randomly sampling known functions 100 times. Applying a *Z* test, we determined a *P* value for each score. Correcting for multiple testing (35), we obtained functional predictions for 299 proteins (false-discovery rate [FDR], <0.05). The heat map in Fig. 3 shows the range of functions predicted for these proteins, including 60% predicted to be involved in transcriptional and translational activities. In Table S4, we present the functional profiles of all proteins in the order in which they appear in Fig. 3. The s-index value increased for 55% of proteins when their functions were predicted with the augmented network. As shown in Fig. 1D, the augmented network neighborhood of SP_1876 suggested that the majority of network neighbors revolved around transcriptional functions (Table 1). As indicated in Fig. 3, we confirmed this observation, as we predicted an involvement of SP_1876 in transcriptional functions with a probability value of 0.66 (Table S4).

10.1128/mSystems.00019-17.4 TABLE S4  Prediction of functional classes of 299 poorly characterized proteins in *S. pneumoniae* by the use of the meta-interactome. Prediction scores represent the probability of a certain function as featured in Fig. 3 that were significant on an FDR = <0.05 basis. Download TABLE S4, XLSX file, 0.1 MB. Copyright © 2017 Wuchty et al. 2017 Wuchty et al. This content is distributed under the terms of the Creative Commons Attribution 4.0 International license.

## DISCUSSION

Genome-wide protein interaction maps have guided functional analyses for several bacterial species, with the notable exception of Gram-positive species, such as *B. subtilis* and *S. pneumoniae*. Here, we provide the first large-scale study of protein-protein interactions in *S. pneumoniae*. While streptococci, including *S. mutans*, *S. pyogenes*, *S. sanguinis*, and others, are important pathogens (36), none of those species have been systematically investigated for protein-protein interactions. While these species have diverged significantly, they still share a core proteome of about 60% to 70% of their proteins (37), indicating that a large fraction of interactions in *S. pneumoniae* are likely conserved in other streptococci. Given that genetic diversity within the genus is considerable, including a vast pan-genome (38), we expected that thousands of proteins would remain uncharacterized within the group. In fact, among the 2,045 PPIs that we found in our study, 1,328 involved at least one uncharacterized protein, while 195 interactions had two uncharacterized proteins.

Even though the majority rule is a powerful tool for predicting protein function from interaction data, the number of available functional annotations limits its practicability. Therefore, we augmented our experimentally determined protein interaction network with a bacterial meta-interactome. Such a network was defined as the pool of all interactions between evolutionarily conserved proteins in bacteria for which interaction data were available. Specifically, we accounted for all interactions in other bacteria if at least one protein had an ortholog in *S. pneumoniae*. The meta-interactome included potentially conserved protein interactions that could be used to predict protein function in *S. pneumoniae*. In addition, we show that the functional annotation of even a nonconserved protein that interacts with a protein conserved in *S. pneumoniae* can still contribute to the functional prediction of the conserved protein. The auxiliary interactions boosted the strength of the prediction method, allowing us to assess the function of 342 *S. pneumoniae* proteins and to assign possible functions to 299 of these. As a consequence, the consideration of interologous interactions significantly improved our ability to predict functions of uncharacterized proteins, annotations that can be used as a launching point for detailed functional analyses in both *Streptococcus* species and other bacteria.

Our report provides numerous hypotheses for future analysis of protein function in *S. pneumoniae* and other Gram-positive bacteria and likely many for Gram-negative species as well. However, expert evaluation of our predictions is necessary in order to design follow-up experiments and detailed functional analyses.

## MATERIALS AND METHODS

### Detection of protein interactions in *S. pneumoniae*.

We used a two-step approach to study protein-protein interactions (PPIs) in *S. pneumoniae*. The first step was a global screen of pairwise interactions among all available (~1,704) open reading frames using a Gal4-based yeast two-hybrid (Y2H) system. In the second step, we focused on proteins of unknown function, using a LexA-based Y2H system. On the basis of ample previous experience (23–25), we know that the sets of interactions detected by different Y2H systems overlap only partially; hence, this approach promised better interactome coverage than a single-step system.

### Bait and prey libraries.

In the Gal4-based Y2H screen, full-length ORFs of *S. pneumoniae* were shuttled from pENTR221 entry clones (Pathogen Functional Genomics Resource Center [PFGRC]; formerly at the J. Craig Venter Institute [JCVI], Rockville, MD; now maintained by BEI]) into Y2H bait plasmid pDEST22 (Invitrogen, Carlsbad, CA) using Gateway cloning.

Individual bait plasmids were transformed into haploid yeast strain CG-1945 and prey plasmids into Y187 (Clontech, Mountain View, CA) as described previously (39).

The prey library was created by growing all plasmid strains of the *S. pneumoniae* entry clone library individually in selective Luria Broth (LB) medium, followed by pooling and plasmid isolation. The resulting entry clone plasmid pool was shuttled into the pGADT7g and pDEST22 prey plasmids by the use of a Gateway LR reaction (Invitrogen). The reaction mixture was then transformed into electrocompetent *E. coli* DH10B (ElectroMAX; Invitrogen) and grown in selective LB medium, and the plasmids were isolated. Plasmid pools were then transformed into Y187 as described previously (40) and spread onto 24-by-24-cm dishes containing Synthetic Defined (SD) agar. Finally, all colonies were scratched from the plates, resuspended in 25% glycerol, and stored as 50-μl aliquots at −80°C.

### Mating.

Yeast bait and prey libraries were grown and mated as described in reference 41 with the following adjustments. For liquid mating, corresponding volumes of each individual bait strain and the prey pool, at an optical density at 600 nm (OD_600_) of 2, were mixed. Selection of Y2H positive diploids was carried out on plates (15-cm diameter) containing agar medium with SD medium (MP Biomedicals, Solon, OH) without the amino acids Leu, Trp, and His and supplemented with 0.1 mM 3-amino-1,2,4-triazole (3-AT). The screening plates were incubated for 3 to 5 days at 30°C. To check the mating efficiency, a 1:10,000 dilution was plated on -Leu-Trp SD agar in parallel to the screens and the number of diploid colonies was determined. A screen was repeated if the number of colonies was <200,000. For autoactivating baits, screens were repeated on 1 mM and 10 mM 3-AT.

Y2H-positive preys were identified by colony PCR after zymolyase (amsbio) treatment using BIOTAQ Red DNA polymerase (Bioline) following enzymatic purification performed as described in reference 42. PCR products were verified by agarose gel electrophoresis and analyzed by Sanger sequencing (GATC, Cologne, Germany). The identities of the sequences were confirmed by BLASTN analysis. The sequences were subjected to blast analysis against a sequence database with ORF sequences of *S. pneumoniae* strain TIGR4 (control).

In the LexA-based Y2H screen, independently of the Gal4 system described above, we used the LexA-based yeast two-hybrid system (43) to screen specifically for proteins that interact with *Streptococcus pneumoniae* proteins that were annotated as “hypothetical.” We started with the same 1,297 sequence-validated *Streptococcus pneumoniae* Gateway entry clones described above (constructed by the PFGRC at JCVI). We successfully subcloned 1,219 of these open reading frames (ORFs) into activation domain (AD) vector pJZ4attR (44) and created an array of yeast clones (the AD array) in 96-well plates (see Table S1 in the supplemental material). We also subcloned 1,005 of the sequence-verified ORFs into LexA DNA-binding domain (BD) vector pNLex(NLS)attR (44) and created an array of these yeast clones (BD array) (Table S2). The BD array included 360 clones for *S. pneumoniae* genes annotated as encoding a “hypothetical protein” or “conserved hypothetical protein.” We used these 360 BD clones to screen the entire AD array using a modified two-phased pooled mating approach (1, 45). Briefly, we mated each of the individual BD clones with an array of AD clone pools, where each position of the array contained a pool of 8 AD clones taken from one column of the original AD array; the AD clone pools were arrayed on two 96-well plates. In cases in which the reporters were active with a particular BD clone and an AD pool, the BD was mated with the 8 individual AD clones to determine the interaction. Confidence scores were then assigned to the interactions (1), and the 1,771 interactions with scores of >0.5 were retested. A total of 1,513 gave positive results. After filtering out interactions with very low reporter scores or involving proteins that activated the reporters on their own, we identified 1,054 interactions; 994 of these were then tested in the opposite orientation, with the BD and the AD swapped. A total of 242 (24.3%) were detected in the opposite orientation.

### Determination of orthologous sequences.

Utilizing all-versus-all BLASTP searches with the InParanoid script (29) in protein sets of two species, sequence pairs with mutually best scores were selected as central orthologous pairs. Proteins of both species that showed such an elevated degree of homology were clustered around these central pairs, forming orthologous groups (OGs). The quality of the clustering was further assessed by a standard bootstrap procedure. We considered only the central orthologous sequence pair with a confidence level of 100% to be the real orthologous relationship. Protein sequence information in the bacterial organisms was retrieved from UniProt (46).

### Protein interaction data.

We used 2,231 binary interactions between *E. coli* proteins that we had determined through yeast two-hybrid screens previously (6). As for the other yeast two-hybrid screen sets, we utilized 12,012 interactions in *C. jejuni* (1), 3,121 interactions in *M. loti* (5), 3,236 interactions in *Synechtocystis* sp. strain PCC6803 (3), 2,907 interactions in *H. pylori* (11), 3,684 interactions in *T. pallidum* (2), 783 interactions in *B. subtilis* (12), and 8,042 interactions in *M. tuberculosis* (4). The bacterial meta-interactome was created by mapping the proteins of these species to orthologous groups (OGs) from EggNOG (24) and then merging all interactions among these OGs into a single network.

### Functional prediction of unknown proteins in *S. pneumoniae*.

We modeled the prediction of a functional class σ of a protein *i* as a Potts model (19). In particular, we considered functional annotation of proteins in *S. pneumoniae* using Clusters of Orthologous Group (COG) classes, i.e., functional annotations derived from the EggNOG database (24). All proteins without a functional annotation as well as proteins that were either classified as “unknown” or had a “general” function (such as “membrane protein” or “ABC transporter”) were randomly assigned a function corresponding to the remaining 23 classes. In particular, we minimized the global function $E=-\sum_{i,j}J_{ij}\text{δ}(\sigma_{i},\sigma_{j})-\sum_{i}h_{i}\left(\sigma_{i}\right)$, where *J*_*ij*_ is the adjacency matrix of the interaction network that accounts for unclassified proteins. In particular, *J*_*ij*_ = 1 if unclassified proteins *i* and *j* interact and vice versa. δ(*i, j*) is the discrete δ function, where δ = 1 if unclassified proteins *i* and *j* have the same function (i.e., σ_*i*_ = σ_*j*_) and vice versa. As a consequence, the first term allows us to optimize the number of interactions between unclassified proteins if they are predicted to have the same function. Depending on the function assigned to an otherwise unclassified protein, the second term aims to optimize support for the assigned function of protein *i*. In particular, we determine the number of classified proteins *h*_*i*_(σ_*i*_) that interact with unclassified protein *i* with the same function σ that was assigned to unclassified protein *i*.

To minimize *E*, we applied a simulated annealing approach that features an effective temperature *T*. After initially assigning random functions to all unclassified proteins, we randomly selected a protein, changed its function to a different class, and determined the energy of the new configuration. If the difference of energies Δ*E* = ≤0, the new configuration was accepted. If Δ*E* = >0, the new configuration was accepted with probability *p* = *e^−ΔE/T^*. To obtain stabilized functional configurations, we repeated such a Monte Carlo step 10,000 times (19). Subsequently, we increased the inverse of *T* by 0.01 in each step and repeated such Monte Carlo steps. Since minimum energy solutions are not unique, we repeated such runs of simulated annealing 100 times and considered the fraction of times that an unclassified protein *i* was observed in a certain functional state σ to be an estimate of the probability that protein *i* belongs to class σ.

### Transitions between functional classes.

We randomly sampled 20% of all functionally annotated proteins in *S. pneumoniae* 1,000 times and utilized the remainder to predict the functions of the sampled proteins in the protein interaction network of *S. pneumoniae* as well as in the augmented network. Each gene in the sample was assigned the most probable function. As such, we determined *n*_*i→j*_, the number of times that the original gene in the network of *S. pneumoniae* was predicted to have function *i* while its function changed to *j* in the augmented network. For each transition from function *i* to *j*, we determined its probability $p_{o}\left(i\to j\right)=\frac{n_{i\to j}}{N}$, where *N* is the total number of genes that were considered. For a null model, we determined an expected probability of transitions from function *i* to *j* $p_{e}\left(i\to j\right)=\frac{n_{i\to }n_{\to j}}{N^{2}}$. Specifically, *n*_*i*→_ is the number of times that genes were found to have function *i* in the original protein interaction network of *S. pneumoniae*, while *n*_→*j*_ is the number of times that genes were found to have predicted function *j* in the augmented network. Combining these probabilities, we determined a log-odds ratio $r=\frac{p_{o}{\left(1-p_{o}\right)}^{-1}}{p_{e}{\left(1-p_{e}\right)}^{-1}}$. For large samples, we estimated the variance of the odds distribution as $\sigma^{2}=n_{ij}^{-1}+{\left(N-n_{ij}\right)}^{-1}+a^{-1}+{\left(b-a\right)}^{-1}$, where *a* = *n*_*i*→_*n*_→*j*_ and *b* = *N*^2^. We calculated a *P* value for the significance of a link between two classes by a *Z* test, where $Z=\frac{r}{\sigma }$, and considered each link with *P* = <0.05.

### Heterogeneity of functional prediction.

The Simpson s-index considers the fractions with which a given protein was assigned to a functional class. In particular, we calculated its heterogeneity of functional fractions as a Simpson diversity (34) index defined as $s=\overset{N}{\underset{i=1}{\sum }}p_{i}^{2}$, where *p*_*i*_ is the fraction with which a given protein was assigned to functional class *i*. Such a measure tends to reach a value of 1 if one function dominates the distribution of fractions and vice versa.

### Enrichment of accuracy as a function of degree.

To compare the prediction results that we obtained with the original network and the augmented network of protein interactions in *S. pneumoniae*, we calculated the fraction of correctly predicted functions in bins of proteins with a given number of interaction partners in the original network of *S. pneumoniae*. Since each protein was assigned to a functional class with a certain probability, we labeled each protein with the most probable function. We defined the enrichment of accuracy in a given bin of degree *k* as $s=\overset{N}{\underset{i=1}{\sum }}p_{i}^{2}$, where *f*_*k*_ is the fraction of correctly predicted functions of proteins with degree *k* in the original network of *S. pneumoniae*. In turn, *f*_*k*,*m*_ reflects the rate of correctly predicted functions using the augmented network.
